# Supplementary material for: WordCommentsAnalyzer: A windows software tool for qualitative research
Source: F1000Res. 2018 Sep 4;7:536. Originally published 2018 May 3. [Version 2] doi: 10.12688/f1000research.14819.2 (PMC6137411; doi:10.12688/f1000research.14819.2)
Supplement: Supplementary file 3 [file f1000research-7-17614-s0002.tgz › b2a79496-4ada-47c4-9682-a99ce867836d.zip!/Eight Iranian Health Journals/The Journal of Toloo-e-Behdasht_6.docx]

© The Journal of Toloo-e-Behdasht. This work is licensed under CC BY 4.0 (https://creativecommons.org/licenses/by/4.0/).

Volume & Issue: دوره 16 - شماره 2

Title: بررسی اثر پرتوالکترونی بر روند تولید آفلاتوکسین B1در پسته های تلقیح شده با آسپرژیلوس فلاووس

http://tbj.ssu.ac.ir/article-1-2042-fa.html

مقدمه: پسته  یک محصول مهم صادراتی در برخی از کشورها به ‌ویژه ‌ایران است. پسته‌ در بین محصولات کشاورزی با بالاترین خطر آلودگی به آفلاتوکسین قرار دارد. آفلاتوکسین B1 بیشترین پتانسیل سرطان‌زایی را در بین آفلاتوکسین ها دارد. بدون شک معضل اصلی و مهم کشور در عرصه صادرات پسته، مسئله آلودگی پسته به آسپرژیلوس فلاووس و آفلاتوکسین و پیدا کردن راه مناسب جهت پیشگیری از تولید آن است. لذا هدف از این مطالعه تعیین اثر پرتو الکترونی بر روند تولید آفلاتوکسین  B1در پسته های تلقیح شده با آسپرژیلوس فلاووس بود. روش بررسی: مطالعه حاضر به صورت مداخله ای(تجربی) انجام شد. اسپور آسپرژیلوس فلاووس تولید کننده آفلاتوکسین به 10 نمونه  بسته بندی شده پسته، تلقیح گردید. نمونه های پسته در دوزهای 1، 3، 5 و 7 کیلوگری با پرتوالکترونی مواجهه یافتند. سپس نمونه ها در بازه زمانی صفر،210،180،90،60،30 روز در دمای محیط نگهداری گردیده و با نمونه های شاهد از نظر تولید آفلاتوکسین B1 با استفاده از نرم افزار SPSS نسخه 16 توسط آزمون Kruskal Wallis مقایسه شدند. سنجش آفلاتوکسین B1 به وسیله کیت الایزا و با دو بار تکرار انجام گردید. یافته ها: میانگین غلظت آفلاتوکسین در کل نمونه ها 97/59 نانوگرم بر گرم بود. بین میزان تولید آفلاتوکسین در دوزهای مختلف در روزهای صفر،90،60،30 تغییر معنی داری از نظر آماری مشاهده نگردید(05/0<P) ولی در روزهای 180 و 210 دارای سطح معنی بینابینی بود(06/= P). نتیجه گیری: قرار دادن نمونه های پسته در معرض پرتوالکترونی می تواند با از بین بردن قارچ از تولید سم آفلاتوکسین پیشگیری نماید.

Title: بررسی فراوانی کمپیلوباکتر در طغیانهای ناشی از بیماریهای منتقله از غذا درکشور

http://tbj.ssu.ac.ir/article-1-2258-fa.html

مقدمه: بیماری‌های منتقله از غذا یکی از مشکلات گسترده و رو به رشد در جوامع بشری هست. گونه‌های کمپیلوباکتر از مهم‌ترین پاتوژن های عامل گاستروآنتریت های باکتریایی هستند که عموماً از طریق مواد غذایی با منشأ حیوانی منتقل می‌شوند. هدف این مطالعه شناسایی کمپیلوباکتر در نمونه‌های طغیان اسهالی ناشی از بیماری‌های منتقله از غذا در سطح کشور است. روش بررسی: این مطالعه مقطعی-توصیفی در بهار و تابستان سال 1394 بر روی 102 طغیان ناشی از غذا که شامل تعداد 305 نمونه سوآپ مدفوع افراد مبتلا به اسهال از استان‌های مختلف ایران بود، صورت گرفت. تمامی نمونه‌ها ازنظر کشت میکروب کمپیلوباکتر موردبررسی و نتایج مطالعه با آنالیز توصیفی، آزمون کای دو و با استفاده از نرم‌افزار SPSS21 صورت پذیرفت. یافته‌ها: از کل 102 طغیان غذایی ، بیشترین موارد مربوط به استان زنجان با 25 طغیان (5/24%) واستان یزد با 70 نمونه اسهال( 23% ) بیشترین موارد را گزارش کردند. از 305 مورد اسهالی 119 مورد (39%) مربوط به غذا، 35 مورد (5/11%) ناشی از آب و 151 مورد (5/41%) نامشخص بود (001/0>P). از کل طغیان‌ها دو طغیان از استان یزد آلوده به کمپیلوباکتر از نوع کمپیلوباکترکلی بودند. علائم بالینی بیماران شامل اسهال معمولی (9/30%)، کرامپ شکمی (5/68%)، تب (8/31%)، سردرد (3/42%)، اسهال خونی (2/5%)، تهوع (3/62%) و استفراغ (9/64%) بود. نتیجه‌گیری: نتایج بدست آمده نشان داد که گونه کمپیلو باکتر کلی بر خلاف گذشته که گونه ژژونی به عنوان عامل اسهال شناخته شده بود،در این تحقیق به عنوان عامل اسهال در طغیانهای غذایی کشور معرفی گردید.

Title: استفاده از عوامل اکسنده با استفاده از تکنیک فراصوت-الکتروشیمی و روش آماری CCD

http://tbj.ssu.ac.ir/article-1-1410-fa.html

مقدمه:یکی از آلاینده‌های مهمی که محیط زیست بشر را مورد تهدید قرار داده‌اند، ترکیبات رنگی می‌باشند. بر این اساس تاکنون روش‌های متعددی برای حذف این آلاینده‌ها از پساب صنایع به ویژه صنعت نساجی ارائه شده‌اند. یکی از این روشهایی که در چند سال اخیر مورد توجه قرار گرفته‌اند، روش سونوالکتروشیمی به شمار می‌آید.   روش بررسی: این مطالعه بنیادی-کاربردی است . در این کار با استفاده از روش آماری CCD اثرات متقابل چهار متغیر مهم pH محیط، غلظت محلول رنگی، مدت زمان فرآیند و پتانسیل در رنگبری از پساب ترکیب رنگی بازیک بلیو47 (BB47) مورد بررسی و ارزیابی قرار گرفت. یافته‌ها: بر اساس آزمایش‌های صورت گرفته و نتایج حاصل بدون حضور عامل اکسنده هیدروژن پراکسید، بهترین شرایط برای رنگبری در شرایط بهینه 9=pH، غلظت رنگ µM 303/3،زمان تماس min 93،پتاسیل V0/81 بدست آمد. از سوی دیگر مدل ارائه شده توسط نرم‌افزار با پیش‌بینی میزان رنگبری برابر % 92/8با آزمایش‌های تجربی مورد ارزیابی قرار گرفت که میزان رنگبری % 92/34به صورت تجربی مدل ارائه شده توسط نرم‌افزار را تایید می‌نماید. حذف COD با توجه به شرایط بهینه مذکور توسط فرآیند ترکیبی فراصوت-الکتروشیمی %96 بوده است. نتیجه‌گیری: CCD یک تکنیک خوب برای بررسی و مطالعه تاثیر همزمان متغیرهای مهم بر حذف رنگ BB47 مورد استفاده قرار گرفت. بر این اساس شرایط بهینه (9=pH، غلظت رنگ µM 303/3، زمان تماتس min93، پتاسیل V0/81) توسط نرم‌افزار تعیین گردید. درصد رنگبری پیش‌بینی شده توسط مدل در این شرایط%92/8 بود که پس از انجام آزمایش مقدار تجربی %92/34بدست آمد که نزدیکی پاسخ‌ها نشان‌دهنده‌ی مناسب بودن مدل می‌باشد.

Title: حذف فسفر توسط پوکه معدنی خام و پوکه معدنی اصلاح شده با پراکسید هیدروژن

http://tbj.ssu.ac.ir/article-1-1477-fa.html

چکیده مقدمه: فسفر به عنوان یکی از عوامل اصلی محدود کننده در استفاده مجدد از فاضلاب و همچنین یکی از عناصر مهم در بروز پدیده اتروفیکاسیون در منابع آبی می باشد. لذا، پژوهش حاضر به منظور بررسی کارائی پوکه معدنی و شکل اصلاح شده آن با پراکسید هیدروژن در حذف فسفر از محلول های سنتیتیک انجام گرفته است. روش بررسی: این مطالعه یک مطالعه بنیادی-کاربردی می باشد که در سیستم ناپیوسته انجام گرفت. دراین مطالعه، پوکه معدنی‌خام و شکل اصلاح‌شده آن در جهت حذف فسفر مورد استفاده قرار گرفت. پارامترهای مختلفی از قبیل pH محلول (2-10)، غلظت جاذب (g/L 10-2)، غلظت اولیه فسفر (mg/L 20-5)، قدرت یونی محلول و زمان تماس مورد بررسی قرار گرفت. آزمایشات در سیستم ناپیوسته و با تغییر دادن یک پارامتر و ثابت نگه داشتن سایر پارامترها انجام گرفت. یافته ها: نتایج  نشان داد که پراکسید هیدروژن کارایی پوکه معدنی را بدون تغییر در ساختار آن ارتقا می دهد. بیشترین میزان حذف فسفر در دوز جاذب g/L 10، زمان تماس 130 دقیقه، pH برابر 6 و غلظت mg/L 20 از فسفر اتفاق می‌افتد. در این شرایط بیش از 69% و 97% از فسفر به ترتیب توسط پوکه معدنی خام و شکل اصلاح شده آن حذف گردید. علاوه برآن نشان داده شد که افزایش قدرت یونی محلول باعث کاهش کارایی حذف فسفر می گردد. معادله شبه درجه دوم با ضریب رگرسیون خطی بالایی روند حذف فسفر را توسط جاذب های مورد مطالعه نشان داد. بحث و نتیجه گیری: نتایج حاصل از این تحقیق نشان داد که پوکه معدنی خام قابلیت خوبی در حذف فسفر از خود نشان می‌دهد. علاوه براین مشخص گردید که اصلاح ساختار شیمیایی جاذب خام باعث افزایش ظرفیت جذب آن می‎‌گردد.

Title: تأثیرمداخله ی آموزش مبتنی بر الگوی بزنف در ارتقاء رفتارهای پیشگیری کننده از بیماری سالک در بین والدین خانوارهای ساکن در شهرستان رامهرمز در سال 1394

http://tbj.ssu.ac.ir/article-1-2075-fa.html

مقدمه : انجام مطالعه و برنامه مداخله آموزش برای تغییر رفتارها در جهت پیشگیری از بیماری سالک در مناطق آندمیک ضروری است. این مطالعه با هدف اثربخشی آموزش مبتنی بر الگوی بزنف در ارتقاء رفتارهای پیشگیری کننده از بیماری سالک در بین والدین خانوارهای تحت پوشش مراکز بهداشتی درمانی شهرستان رامهرمز طراحی و اجرا شد. روش بررسی :  این مطالعه، به روش مداخله ای بود که در سال 1394 انجام شد، حجم نمونه 154 نفر بود که  از کل مراکز بهداشتی و درمانی رامهرمز  بصورت تصادفی- خوشه ای انتخاب شدند .سپس ازواحدهای مورد پژوهش داده های مورد نیاز با استفاده از ابزار پرسشنامه گرد آوری شد و 2جلسه آموزشی برگزار گردید و پس از 3 ماه از آموزش ، داد ه ها مجدداً جمع آوری شدند ونهایتاً داده ها مورد تجزیه و تحلیل قرار گرفت. یافته ها : میانگین تغییرات نمرات مربوط به سازه های الگوی بزنف شامل آگاهی، نگرش، قصد رفتار، عوامل قادر کننده و رفتار در والدین قبل و بعد از مداخله آموزشی به طور معنی داری افزایش یافت (0001/0>P) ولی میانگین تغییرات نمره سازه هنجارهای انتزاعی قبل و  بعد از مداخله آموزشی در والدین خانوارها تغییرات معنی داری نداشتند(042/0>P). بحث و نتیجه گیری: یافته های این بررسی نشان داد که برنامه آموزشی بر اساس الگوی بزنف، همه جنبه های لازم جهت ایجاد تغییر و ماندگاری رفتار آموزشی در والدین را دارد. و در پایان می توان گفت که اجرای برنامه بر اساس مدل بزنف، برای والدین مفید بوده و تأثیر قابل توجهی را در آنان داشته است.

Title: اختلال هویت جنسیتی و عوامل اجتماعی موثر بر آن در بین دگرجنس خواهان رجوع کننده به مراکز سازمان بهزیستی کشور طی سال های94-1392

http://tbj.ssu.ac.ir/article-1-2390-fa.html

مقدمه:اختلال هویت جنسیتی شدیدترین درجه ملال جنسی است و به احساس ناراحتی از ساخت آناتومیک جنسی خود و آرزوی خلاصی از اعضای تناسلی خویش و زندگی کردن به صورت فردی از جنس مقابل تعریف می‌شود. این اختلال از سنین کودکی بوجود می‌آید و دارای پیامدهای منفی برای افراد در بزرگسالی می‌باشد، هدف از این مطالعه، سنجش اختلال هویت جنسی و نیز بررسی مهمترین عوامل اجتماعی که بر بروز این اختلال موثرند می باشد . روش بررسی:مطالعه حاضر از نوع پیمایشی است که در بین 160 نفر از افراد دارای اختلال هویت جنسیتی مراجعه کننده به مراکز بهزیستی سراسرکشور با استفاده از روش نمونه‌گیری خوشه ای چندمرحله‌ای انجام گرفت. ابزار گردآوری اطلاعات پرسشنامه استاندارد سنجش اختلال هویت جنسیتی است و روایی  پرسشنامه با محاسبه ضریب آلفای کرونباخ برای متغیر اختلال هویت جنسیتی، برابر با 76/0 می‌باشد. توصیف و تحلیل اطلاعات با  نرم افزار spss نسخه19 انجام گرفت. یافته‌ها:نتایج نشان می‌دهد که میانگین اختلال هویت جنسی پاسخگویان برابر با 84/3 ازپنچ است که بیانگر سطح بالای اختلال هویت جنسی در بین پاسخ‌گویان می باشد. هم چنین نتایج نشان داد که بین متغیرهای مورد مطالعه با اختلال هویت جنسی رابطه معنی‌داری وجود دارد، در نهایت اینکه متغیرهای تحقیق به میزان 26 درصد تغییرات اختلال هویت جنسی را پیش بینی می‌کنند. نتیجه‌گیری:بر اساس یافته‌های تحقیق می‌توان امیدوار بود که با آشنا‌سازی خانواده‌ها و افراد مبتلا از طریق منابع رسمی و غیررسمی در ارتباط با عوامل تاثیرگذار( از جمله برچسب زنی، تصحیح اصول تربیت جنسی و کنترل روابط با همسالان غیرهمنجس)به کاهش و درمان وضعیت اختلال هویت جنسیتی کمک کرد.

Title: به‌کارگیری ترم رطوبت در مدل‌سازی دینامیک فرآیند تصفیه هوای آلوده به هگزان

http://tbj.ssu.ac.ir/article-1-2331-fa.html

مقدمه: بیوفیلتراسیون هوای آلوده به ترکیبات فرار آلی یک روش تصفیه بیولوژیکی است که با عبور هوا از درون بستر پرشده، ماده آلاینده از فاز گاز به فاز بیوفیلم منتقل‌شده و توسط میکروارگانیسم­ها تجزیه می­گردد. فعالیت میکروب­های تجزیه­کننده به‌شدت تحت تأثیر رطوبت بستر است به‌طوری‌که رطوبت بستر مهم‌ترین فاکتور برای کنترل عملکرد بهینه بیوفیلتراسیون به‌حساب می­آید. یکی از راهکارهای ارائه‌شده برای کاهش مشکلات خشکی بستر، استفاده از سوپر جاذب‌های آب است که قابلیت ذخیره‌سازی و نگهداری آب را دارند. روش بررسی: برای اولین بار، یک مدل دوبعدی از بیوفیلتراسیون هوای آلوده به هگزان ارائه می­گردد که در آن ترم­های رطوبت بستر و پراکندگی محوری در موازنه انتقال جرم در نظر گرفته شدند. اثرات استفاده از سوپر جاذب آب نظیر افزایش مداوم تخلخل بستر و سطح ویژه آن درون معادلات وارد شدند. برای حل عددی، معادلات به‌دست‌آمده، با روش حجم محدود گسسته سازی شدند. در ادامه جهت تعیین صحت پیش­بینی­های مدل، آزمایش­های بیوفیلتراسیون با دبی L/min 7/0 و غلظت ورودی g/m3 2 انجام گرفت. یافته‌ها: مدل توانست الگویی از افت محتوی رطوبت بستر بعد از قطع آبدهی بستر را ارائه کند. همچنین داده­های آزمایشگاهی افزایش غلظت خروجی آلاینده به دلیل کاهش رطوبت بستر به‌خوبی توسط مدل پیش­بینی شدند (خطای استاندارد= 45/0%) و تخمینی از زمان نهایی ازکارافتادن سیستم ارائه شد. نتیجه‌گیری: نتایج مدل‌سازی نشان داد در نظر گرفتن ترم رطوبت در شرایط نا پایا می­تواند حیطه عملکرد مدل را توسعه دهد و فرایند بیوفیلتراسیون را با تمام پیچیدگی­های آن توصیف نماید.

Title: اثر بخشی آموزش زوج درمانی مثبت نگر بر صمیمیت زناشویی و امید به زندگی زنان عادی شهرستان بافق

http://tbj.ssu.ac.ir/article-1-1656-fa.html

مقدمه: صمیمیت زناشویی یکی از عوامل مؤثر بر ثبات خانواده‌ است. همچنین امید به زندگی می تواند این صمیمت را در زنان پررنگ کند. هدف پژوهش حاضر، بررسی اثر بخشی زوج درمانی مثبت نگر بر افزایش صمیمیت زناشویی و امید به زندگی زنان بود. روش بررسی: طرح پژوهش حاضر نیمه آزمایشی از نوع پیش آزمون- پس‌آزمون با گروه کنترل بود. 30 زن با روش نمونه گیری هدفمندانتخاب و به صورت تصادفی به دو گروه آزمایش و کنترل گمارده شدند.جهت گردآوری داده ها از پرسشنامه های  امید به زندگی اسنایدر و صمیمیت زناشویی واکر استفاده گردید. جهت تجزیه و تحلیل داده ها از  نرم افزار SPSS و آزمون آماری تحلیل کواریانس استفاده شد. یافته ها: یافته ها نشان داد نمرات آزمودنی‌ها در نمره امید به زندگی و صمیمیت  در گروه آزمایش در مقایسه با گروه کنترل تغییر قابل‌ملاحظه‌ای داشته است. نتیجه‌گیری: نتایج بیانگر آن است، زوج درمانی مثبت نگر می تواند برافزایش امید به زندگی و صمیمیت زناشویی تاثیر بگذارد.

Title: کاربرد روش بیزی در بررسی روایی سازه های موازنه تصمیم گیری و خود کارآمدی مدل فرانظریه ای در ارتقا رفتار افراد پیش دیابتی شهر یزد

http://tbj.ssu.ac.ir/article-1-1937-fa.html

چکیده مقدمه: رفتار های تغذیه ای نقش مهمی در پیشگیری ابتلا به دیابت دارند. این مقاله با نگاهی تخصصی به ساختار داده های رتبه ای در مبحث روایی سازه، به معرفی روش بیزی در بررسی روایی سازه های موازنه تصمیم گیری و خودکارآمدی مدل فرانظریه ای جهت بهبود رفتارهای تغذیه ای افراد پیش دیابتی می پردازد. روش بررسی:  این تحقیق یک کارآزمایی تجربی با گروه شاهد است و جامعه پژوهش شامل 220 نفر از افراد پیش دیابتی که در طرح غربالگری شهر یزد شناسایی شده و بالای30  سال داشته، قند خون آنان بین 125 -100 و حداقل پنج کلاس سواد داشتند  می باشد.از نرم افزار  3.2.3 OpenBugs جهت برازش مدل تحلیل عاملی بیزی ترتیبی برای سنجش روایی سازه های خودکارآمدی و موازنه تصمیم گیری مدل فرانظریه ای پروچسکا استفاده گردید. یافته‌ها: تمامی بارهای عاملی سازه های مذکور در سطح معناداری 05/0 معنادار شدند،که این امر روایی سازه ها را تایید می کند . ضریب همبستگی بین سازه های منافع و موانع درک شده معنادر نشد( -0/0763,0/007).به علاوه مدل آماری جهت تحلیل عاملی با داده های رتبه ای ساخته شدکه نسبت شانس و احتمالات حاشیه ای را برای پاسخ به هر گزینه از سوالات مربوط به سازه های ذکر شده برآورد می کند. نتیجه گیری: روش بیزی  با بهره گیری از اطلاعات پیشین مانند: متاآنالیزها و سایر منابع، نسبت به مطالعات انجام شده که از تحلیل عاملی استاندارد و یا سایر روش های تحلیل عاملی داده های رتبه ای استفاده کرده اند حتی با حجم نمونه کمتر، دقت مناسب (از نظرخطای براورد)را دارا می باشد.بنابراین نتایج را می توان در تحقیقات بعدی مورد استفاده قرار داد
